# Supplementary material for: Single catheter strategy for transradial angiography and primary percutaneous coronary intervention enhances procedural efficiency, microvascular outcomes, and cost-effectiveness: Implications for STEMI healthcare in resource-limited settings
Source: PLoS One. 2025 Dec 1;20(12):e0337841. doi: 10.1371/journal.pone.0337841 (PMC12668517; doi:10.1371/journal.pone.0337841)
Supplement: S1 File — (PDF) [file pone.0337841.s002.pdf]

Request for Authorship Change(s)

Authorship change requests are subject to PLOS approval. All applicable sections of this form must be completed in full before we will review your request. Please note that per the PLOS Authorship Policy we require agreement from all authors (including those being added or removed) for any changes to a submission's author list.

PLOS does not generally consider requests to add or remove authors after editorial acceptance; any late-stage requests require approval by the journal's editorial team.

In some cases, depending on the nature, timing, and/or extent of authorship changes requested, PLOS may require verification of the updated author list and contributions by an official at the authors' institution(s). Difficulties reaching an institutional official may delay your submission's peer review or publication.

Section 1. Confirm adherence to PLOS policies

☑ Check here to confirm you have read the *PLOS* Authorship policy, and that the updated author list requested in this form complies in full with the criteria outlined in the Authorship Requirements section of our policy. Key points from the policy that pertain to authorship criteria are summarized here:

> Everyone listed as an author must meet all criteria for authorship, and everyone who meets the criteria for authorship should be listed as an author.
>
> **Authorship criteria for *PLOS Medicine*:**
> 1. Conception and design of the work, acquisition of data, or analysis and interpretation of data; and
> 2. Drafting the article or revising it critically for important intellectual content; and
> 3. Final approval of the version to be published; and
> 4. Agreement to be accountable for all aspects of the work
>
> **Authorship criteria for all other PLOS journals:**
> 1. Substantial contributions to one or more of the following: conception or design of the work; acquisition, analysis, or interpretation of data; creation of new software used in the work; drafting or substantially revising the article; and
> 2. Approved the submitted version (and any substantially modified version that involves the author's contribution to the study); and
> 3. Agrees to be personally accountable for the author's own contributions AND to ensure that questions related to the accuracy or integrity of any part of the work are appropriately investigated, resolved, and the resolution documented in the literature.
>
> Any contributions that do not meet authorship criteria should be discussed in the Acknowledgments section of the manuscript; authors are responsible for obtaining permission to name individuals in the Acknowledgments.
>
> Payment of Article Processing Charges (APC) does not qualify for authorship.

☑ Check here to confirm that all authors (including those to be added or removed) consent to the requested changes.

☑ Check here to confirm that you have read PLOS policies on Competing Interests and Funding Disclosures. Complete the following table to describe any updates needed to your article's disclosures.

	Updates needed to the submission's disclosure statement	No change needed
**Competing Interests**		☑
**Funding**		☑

Section 2. Final manuscript information

**Manuscript number**<br>e.g., PONE-D-17-00000	PONE-D-25-37889R1
**Complete author list, in correct order**<br>Please note any equal contributors with asterisks (*) or hashes (#)	Mohajit Arneja*, Swetharajan Gunasekar*, Dharaneswari Hari Narayanan*, Joshma Joseph, Harilalith Kovvuri, Sharath Shanmugam, Pavitraa Saravana Kumar, Asuwin Anandaram, Vinod Kumar Balakrishnan, Jayanty Venkata Balasubramaniyan, Sadhanandham Shanmugasundaram, Sankaran Ramesh, and Nagendra Boopathy Senguttuvan
**Financial Disclosure** – including any additions/deletions necessary due to the change in authorship	None.
**Competing Interests** – including any additions/deletions necessary due to the change in authorship	The study authors do not have any competing interests to declare.
**Acknowledgments statement**<br>Please acknowledge any removed authors if they contributed to the study in any way, as well as members of any author groups who do not meet our authorship criteria.	We did not remove any authors.

PLOS
A catalyst for better

Section 3. Requests to add authors

Individual author addition #1

**Full name**	Pavitraa Saravana Kumar
**Email address**	pavitraa99@gmail.com
**Full affiliation**	Department of Clinical Research, Sri Ramachandra Institute of Higher Education and Research (SRIHER), Chennai, Tamil Nadu, India
**Briefly describe the reason for adding this author, and explain why they were not included on the submission previously.**	The author, Pavitraa Saravana Kumar, was added as they played an instrumental role in substantially revising the article and provided valuable insights to refining the manuscript based on the peer-review comments. Due their intellectual role and contributions in revising the article, they were included as an author in the revised version after mutual agreement among all other co-authors and corresponding author of the study.

Confirm that this person contributed to **all** of the authorship criteria for the relevant PLOS journal (see above).	✔
**Tick all relevant boxes to indicate this individual's specific contributions:**	
Conceptualization	☐
Data Curation	☐
Formal Analysis	☐
Funding Acquisition	☐
Investigation	☐
Methodology	☐
Project Administration	☐
Resources	☐
Software	☐
Supervision	☐
Validation	☐
Visualization	☐
Writing – Original Draft Preparation	☐
Writing – Review & Editing	✔

Individual author addition #2 (if applicable)

**Full name**	Asuwin Anandaram
**Email address**	asuwin99@gmail.com
**Full affiliation**	Department of Internal Medicine, Lehigh Valley Network, Allenntown, Pennsylvania, United States
**Briefly describe the reason for adding this author, and explain why they were not included on the submission previously.**	The author, Asuwin Anandaram, was added as they played an instrumental role in substantially revising the article and provided valuable insights to refining the manuscript based on the peer-review comments. Due their intellectual role and contributions in revising the article, they were included as an author in the revised version after mutual agreement among all other co-authors and corresponding author of the study.

Confirm that this person contributed to **all** of the authorship criteria for the relevant PLOS journal (see above).	☑
**Specific contributions:**	
Conceptualization	☐
Data Curation	☐
Formal Analysis	☐
Funding Acquisition	☐
Investigation	☐
Methodology	☐
Project Administration	☐
Resources	☐
Software	☐
Supervision	☐
Validation	☐
Visualization	☐
Writing – Original Draft Preparation	☐
Writing – Review & Editing	☑

Author group addition (if applicable)

**Group or consortium name**	Not applicable.
**Author who represents group**	Not applicable.
**Briefly describe the reason for adding this author group, and explain why they were not included on the submission previously.**	Not applicable.

Section 4. Requests to remove authors

Author removal #1

**Full name**	
**Email address**	
**Briefly describe the reason for removing this author at this time given their stated contributions to the manuscript and/or research.**	Not applicable. We have not removed any authors.

**Please fill in all information below for the removed author:**	
Are you updating the article's Acknowledgments to credit this person's contributions?	No
If yes, did they agree to be listed in the Acknowledgments?	No

Please state which authorship criteria have NOT been met by this individual (refer to section 1 of this form), list the specific CREdiT contributions previously attributed to this author and explain why you no longer wish to credit this individual for this work.

Author removal #2 (if applicable)

**Full name**	
**Email address**	
**Briefly describe the reason for removing this author at this time given their stated contributions to the manuscript and/or research.**	Not applicable. We have not removed any authors.

**Please fill in all information below for the removed author:**	
Are you updating the article's Acknowledgments to credit this person's contributions?	No
If yes, did they agree to be listed in the Acknowledgments?	No
**Please state which authorship criteria have NOT been met by this individual (refer to section 1 of this form), list the specific CREdiT contributions previously attributed to this author and explain why you no longer wish to credit this individual for this work.** Not applicable.	

Section 5. Requests for changes to author order

**Prior author list**	Mohajit Arneja*, Swetharajan Gunasekar*, Dharaneswari Hari Narayanan*, Joshma Joseph, Harilalith Kovvuri, Sharath Shanmugam, Vinod Kumar Balakrishnan, Jayanty Venkata Balasubramaniyan, Sadhanandham Shanmugasundaram, Sankaran Ramesh, and Nagendra Boopathy Senguttuvan
**Requested author list**	Mohajit Arneja*, Swetharajan Gunasekar*, Dharaneswari Hari Narayanan*, Joshma Joseph, Harilalith Kovvuri, Sharath Shanmugam, Vinod Kumar Balakrishnan, Jayanty Venkata Balasubramaniyan,
**Briefly describe the reason for requesting a change in author order at this time.**	We have added two new authors as outlined in Section 3. There are no specific changes to author order.

**If any authors' contributions need to be updated, provide the affected authors' names and their updated contributions listings here.** Contributions should be listed using the CRediT Taxonomy terms listed below; list all terms that apply.<br><br>If no changes are needed, enter "N/A" in the response box.	

CRediT Taxonomy for author contributions

- Conceptualization
- Data Curation
- Formal Analysis
- Funding Acquisition
- Investigation
- Methodology
- Project Administration
- Resources
- Software
- Supervision
- Validation
- Visualization
- Writing – Original Draft Preparation
- Writing – Review & Editing
