# Supplementary material for: Single catheter strategy for transradial angiography and primary percutaneous coronary intervention enhances procedural efficiency, microvascular outcomes, and cost-effectiveness: Implications for STEMI healthcare in resource-limited settings
Source: PLoS One. 2025 Dec 1;20(12):e0337841. doi: 10.1371/journal.pone.0337841 (PMC12668517; doi:10.1371/journal.pone.0337841)
Supplement: S2 File — (PDF) [file pone.0337841.s003.pdf]

SRI RAMACHANDRA
INSTITUTE OF HIGHER EDUCATION AND RESEARCH
(Category - I Deemed to be University) Porur, Chennai

From, September 30th, 2025

Dr. Nagendra Boopathy Senguttuvan
Department of Cardiology,
Sri Ramachandra Institute of Higher Education and Research,
Chennai, India

To,

The Editorial Staff and Editor-in-Chief
PLoS One

Dear PLoS One Editorial Members,

We had submitted our Original Research Article, titled "Single Catheter Strategy for Transradial Angiography and Primary Percutaneous Coronary Intervention Enhances Procedural Efficiency, Microvascular Outcomes, and Cost-Effectiveness: Implications for STEMI Healthcare in Resource-Limited Settings" for consideration in PLoS One.

Our manuscript went through the peer review process and we had uploaded the revised manuscript with all changes on September 16th, 2025. Two authors, Ms. Pavitraa Saravana Kumar and Dr. Asuwin Anandaram, played substantial roles in addressing all the comments from the reviewers and they were added as authors during the revision stage. When submitting the revised manuscript, we highlighted these changes in the PLoS One Authorship Change Form.

As requested by the journal staff, we are writing this letter to provide written confirmation from all authors of the study that they approve of the authorship changes.

All e-Signatures are provided below, including that of the newly added authors.

Mohajit Arneja

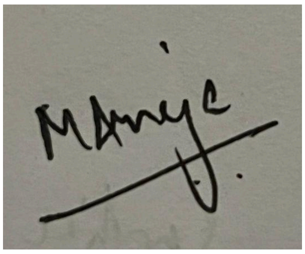

Swetharajan Gunasekar

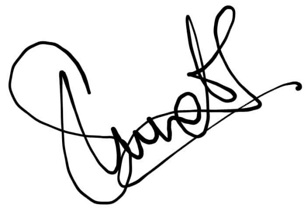

Dharaneswari Hari Narayanan

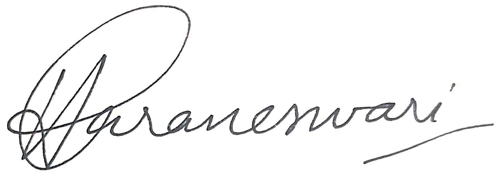

Joshma Joseph

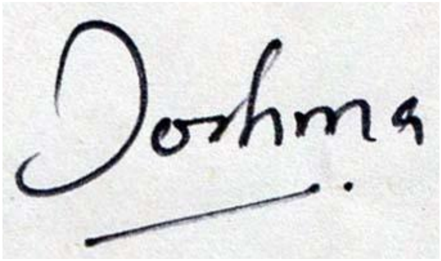

Harilalith Kovvuri

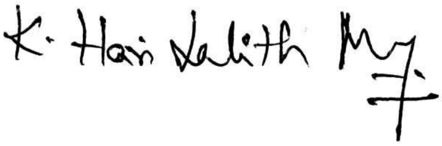

Sharath Shanmugam

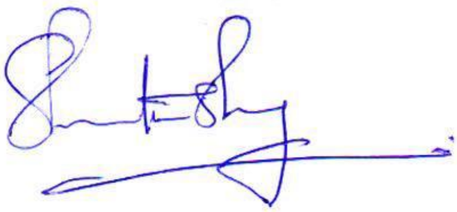

Pavitraa Saravana Kumar **(New Co-Author)**

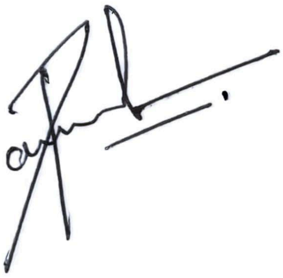

Asuwin Anandaram **(New Co-Author)**

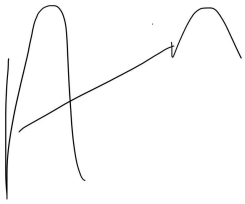

Vinod Kumar Balakrishnan

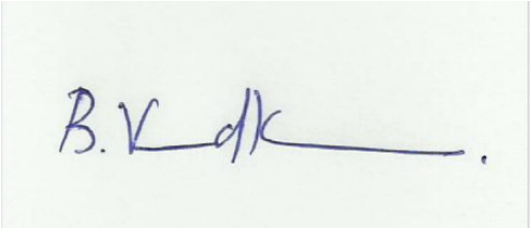

Jayanty Venkata Balasubramaniyan

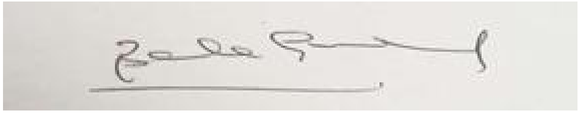

Sadhanandham Shanmugasundaram

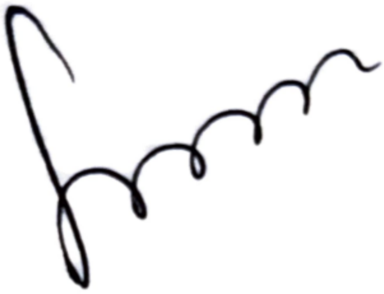

Sankaran Ramesh

Nagendra Boopathy Senguttuvan

We, the duly signed authors above, individually and collectively approve of the authorship changes in acknowledgement of the contributions of Ms. Pavitraa Saravana Kumar and Dr. Asuwin Anandaram to the revision of our manuscript.

Sincerely,

Dr. Nagendra Boopathy Senguttuvan
Professor of Cardiology,
Clinical Lead, Structural Heart Disease Interventions,
Sri Ramachandra Institute of Higher Education and Research,
Chennai, Tamil Nadu, India,
Email: drsnboopathy@gmail.com
